# Intelligent electromagnetic navigation system for robot-assisted intraoral osteotomy in mandibular tumor resection: a model experiment

**DOI:** 10.3389/fimmu.2024.1436276

**Published:** 2024-07-25

**Authors:** Zhijie Zhao, Yichi Zhang, Li Lin, Wenyi Huang, Can Xiao, Jiannan Liu, Gang Chai

**Affiliations:** ^1^ Department of Plastic and Reconstructive Surgery, Shanghai Ninth People's Hospital, Shanghai JiaoTong University School of Medicine, Shanghai, China; ^2^ Department of Stomatology, the First Affiliated Hospital of Soochow University, Suzhou, China; ^3^ National Center for Translational Medicine (Shanghai) SHU Branch, Shanghai University, Shanghai, China; ^4^ Department of Oral and maxillofacial Head and neck Oncology, Shanghai Ninth People’s Hospital, Shanghai Jiao Tong University School of Medicine; College of Stomatology, Shanghai Jiao Tong University; National Center for Stomatology; National Clinical Research Center for Oral Diseases; Shanghai Key Laboratory of Stomatology; Shanghai Research Institute of Stomatology, Shanghai, China

**Keywords:** mandibular tumor, surgical robot, electromagnetic navigation system, injury repair, intelligent planning

## Abstract

**Background:**

Mandibular tumor surgery necessitates precise osteotomies based on tumor boundaries; however, conventional osteotomies often lack accuracy in predicting osteotomy positions and planes, potentially leading to excessive resection of normal bone tissues or residual tumors, thus compromising postoperative quality of life and clinical outcomes. Robotic-assisted surgery (RAS) augmented with artificial intelligence (AI) offers precise localization capabilities, aiding surgeons in achieving accurate osteotomy positioning. This study aimed to evaluate the feasibility and accuracy of a robotic magnetic navigation system for positioning and osteotomy in an intraoral surgical trial of a mandibular tumor model.

**Methods:**

Patient computed tomography (CT) imaging data of mandibular chin and body tumors were utilized to create 3D printed models, serving as study subjects for mandibular tumor resection. Ten pairs of models were printed for the experimental and control groups. The experimental group (EG) underwent osteotomy using a robot-assisted surgical navigation system, performing osteotomy under robotic navigation following alignment based on preoperative design. The control group (CG) underwent traditional surgery, estimating osteotomy position empirically according to preoperative design. Postoperative CT scans were conducted on both models, and actual postoperative results were compared to preoperative design. Osteotomy accuracy was evaluated by positional and angular errors between preoperatively designed and actual osteotomy planes.

**Results:**

For ten randomly selected spots on the left and right sides, respectively, the EG group had mean distance errors of 0.338 mm and 0.941 mm. These values were obtained from the EG group. In the EG group, on the left side, the mean angular errors were 14.741 degrees, while on the right side, they were 13.021 degrees. For the 10 randomly selected spots on the left and right sides, respectively, the CG had mean distance errors of 1.776 mm and 2.320 mm. This is in contrast to the results obtained by the EG. It was determined that the left side had a mean angle error of 16.841 degrees, while the right side had an error of 18.416 degrees in the CG group. The above results indicated significantly lower point errors of bilateral osteotomy planes in the experimental group compared to the control group.

**Conclusion:**

This study demonstrates the feasibility of electromagnetic navigation robot-assisted intraoral osteotomy for mandibular tumors and suggests that this approach can enhance the precision of clinical surgery.

## Introduction

The mandible is the only bone in the face’s bottom region capable of movement. It is pivotal for essential oral functions such as chewing, speaking, and maintaining proper bite alignment. Tumors affecting the mandible can arise as primary growths or secondary manifestations, posing significant challenges to cosmetic appearance and functional capacity within the craniofacial region. The growth and invasion of these tumors can result in pronounced cosmetic deformities and impairments in masticatory function, thus profoundly impacting the overall prognosis and quality of life for affected individuals (1). Furthermore, managing mandibular tumors imposes substantial economic burdens on society and the healthcare system (2–4). Repairing tissue injuries within the mandibular region is intricate and multifaceted, involving a complex interplay of cellular and molecular mechanisms. These mechanisms undergo dynamic regulation across various stages of healing. Traditional approaches to mandibular tumor resection often entail extensive surgical intervention, leading to sizable incisions, prolonged postoperative recovery periods, and a notable decline in the quality of life for patients in the aftermath of surgery. Consequently, achieving optimal outcomes in mandibular tumor surgery necessitates the implementation of precise surgical techniques. Without advanced technologies such as computer-assisted surgery, surgeons historically relied on preoperative computed tomography (CT) scans and their clinical expertise to delineate the optimal osteotomy line. However, making sure that the bone is correctly positioned along the planned osteotomy line before surgery has been a persistent problem when osteotomies are done through intraoral incisions for mandibular tumors. Still, recent improvements in surgical navigation systems and computer-guided techniques have made mandibular tumor surgery more accurate and predictable, leading to better cosmetic and functional outcomes for patients.

With advancements in imaging and computer technology, preoperative three-dimensional reconstruction of mandibular tumors using CT and MRI combined with techniques such as radiomics and deep learning has enabled the operator to locate the boundaries of tumor invasion (5, 6) accurately. However, applying this valuable imaging information to the osteotomy during surgery poses challenges. Traditional surgical methods rely heavily on the operator’s clinical experience and skill, which increases the risk of deviating from the preoperative resection boundaries and makes it difficult to precisely control the angle of the osteotomy plane. As a result, achieving precise osteotomies and optimal reconstruction outcomes post-surgery becomes challenging. Surgical robots, known for their precision, stability, and efficiency, are widely used in various fields, including endoscopic surgery, orthopedics, neurosurgery, and otolaryngology. Combined with artificial intelligence technology (7–9), robots can intelligently assist in preliminary surgical planning, while their precise positioning capabilities aid surgeons in performing osteotomies (10).

In recent years, robot-assisted surgical technology has rapidly developed and expanded into more fields due to its high accuracy, stability, and ability to reduce surgical fatigue among operators (11). Previous studies have highlighted robot-assisted craniomaxillofacial surgery’s effectiveness, accuracy, and safety. However, the prevalent optical navigation systems in this field are limited by light. They cannot fulfill the requirements for intraoral osteotomy line positioning and guided osteotomies in patients with mandibular tumors. Ensuring precise alignment of robot coordinates and target position with intraoperative dynamic tracking and guidance poses a primary challenge in the clinical application of craniomaxillofacial robotic surgery. Among the latest navigation systems introduced in the craniomaxillofacial robotics field, the electromagnetic navigation system stands out for its compact size, freedom from light interference, high positioning accuracy, and real-time tracking and guidance capabilities for the operator (12). Nonetheless, studies confirming the feasibility and accuracy of the electromagnetic navigation system for mandibular tumors in intraoral incisional osteotomies are yet to be conducted.

Building upon prior research, we conducted a model surgical experiment to assess the feasibility and navigational accuracy of electromagnetic navigation robot-assisted intraoral osteotomy for mandibular tumors. Our experimental setup included a custom magnetic surgical navigation robot comprising a UR5 robotic arm, a computer base, and an electromagnetic navigation system (Aurora V3, NDI) to align occlusal tablets automatically. Additionally, we utilized a self-designed metal template. To evaluate the potential clinical application of this system, we analyzed the position of the osteotomy surface based on measurements obtained from model surgical CT scans.

## Methods

### Ethical approval

All procedures involved in the fabrication of the experimental model were conducted in compliance with ethical standards and guidelines. Approval for the experimental protocols was obtained from our independent ethical committee (Approval No. SH9H-2020-T267-3), and all methods were performed under relevant regulations and guidelines.

### Surgical robot based on magnetic navigation localization

**Figure 1:**
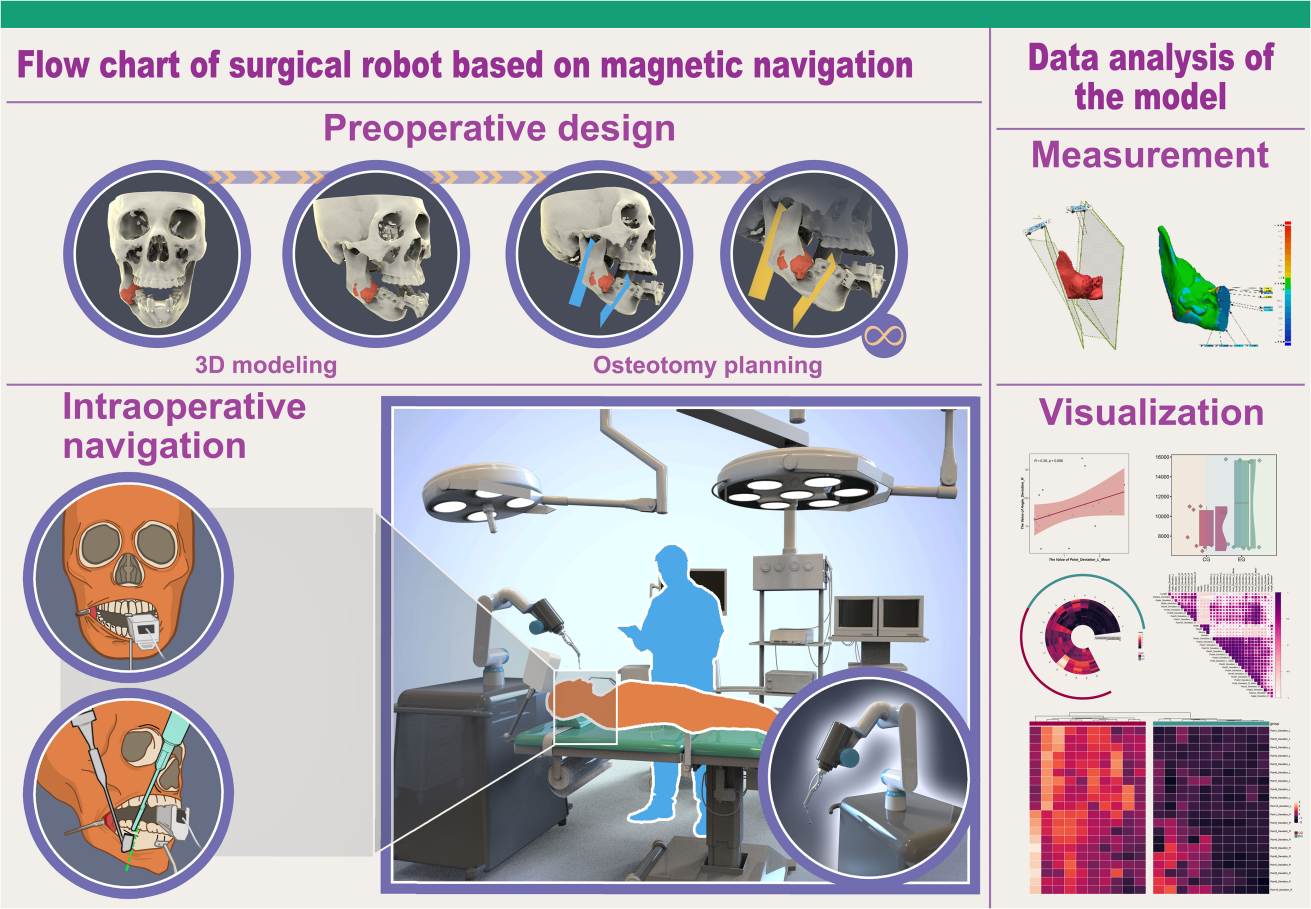
Flow chart of surgical robot based on magnetic navigation.

**Figure 2:**
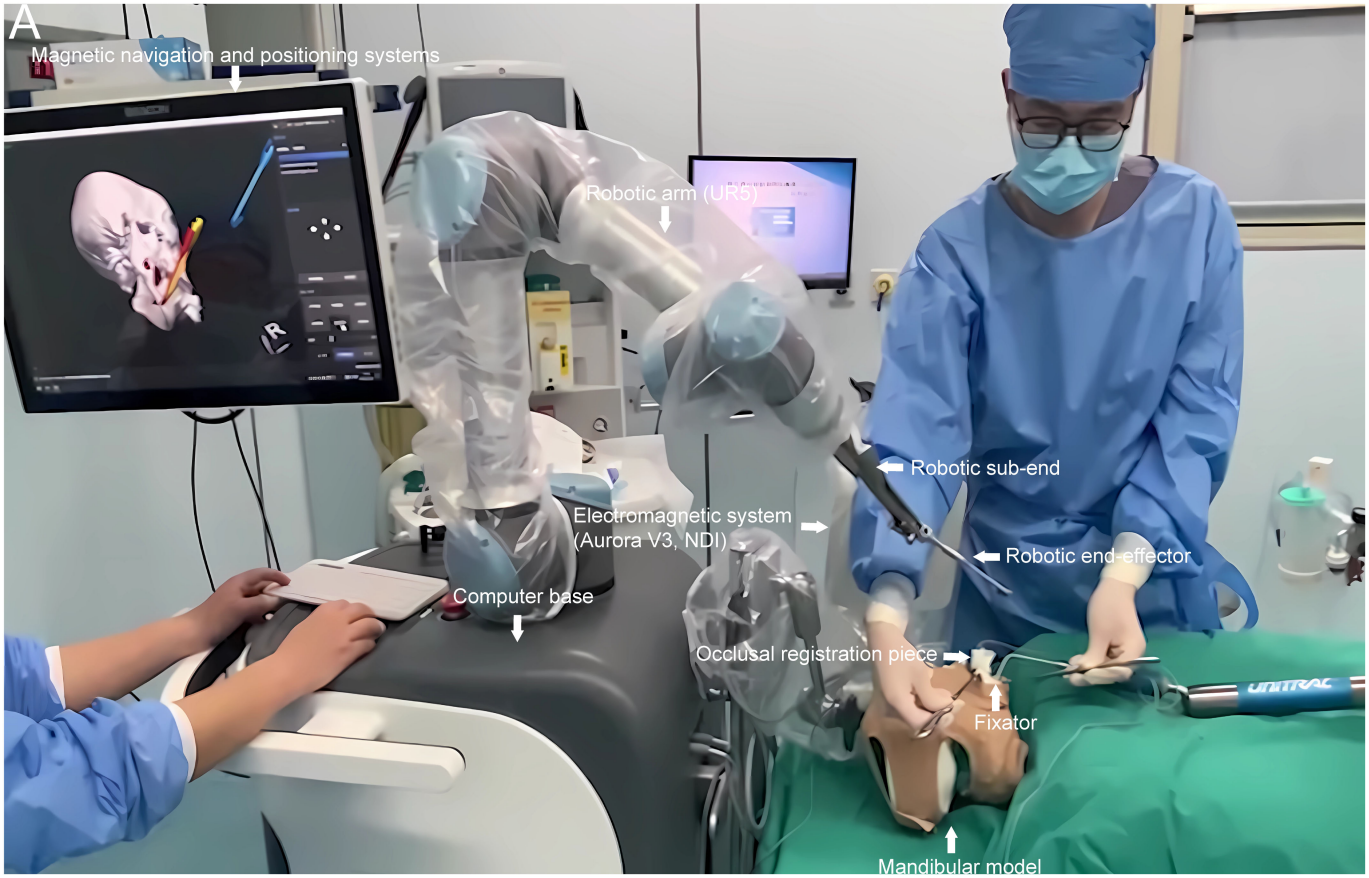
The magnetic navigation surgical robot was utilized in this study. Equipped with a magnetic navigation and localization system, the robot was deployed in an environment conducive to standard model surgical experiments. It primarily comprised a computerized base, a UR5 robotic arm, end fittings attachable to the arm, an electromagnetic navigation system (Aurora V3, NDI), and other essential components. The robot’s navigation and localization systems were tailored for cranial and maxillofacial surgery. A brown headgear simulated the mandibular tumor model’s surgical environment during model surgical experiments, with an occlusal registration piece affixed to the fixator.

The surgical robot employed in this study comprised several components: a computerized workbench, a UR5 robot, an end-effector for the robotic arm, surgical navigation software, and an electromagnetic navigation and positioning system (Aurora V3, NDI) (see **Figures 1**, **2**). The robot assembly included the robot controller and arm, which possessed six degrees of freedom. Each joint of the robotic arm was driven by a motor and moved to the predetermined surgical position as controlled by the computerized workbench’s control commands, allowing for precise intraoperative positioning. Throughout the movement, the surgical navigation software displayed the real-time position of the end-effector. It halted movement upon reaching the planned position, locking the joints to maintain the current position. During intraoperative procedures such as osteotomy, the end-effector of the robotic arm could be substituted with a different subtend guide. The surgical navigation software facilitated preoperative surgical path planning, data collection from the robotic arm and electromagnetic navigation system, and control of the robotic arm’s movement. The electromagnetic navigation system employed fields to localize magnetic marker points, enabling real-time three-dimensional spatial measurements even under occlusion and establishing a spatial mapping transformation between the image and the robotic arm.

### Fabrication of the mandibular tumor model

The experimental model of mandibular tumors was constructed using 3D-CT data obtained from patients with mandibular tumors who were treated at our hospital. The 3D-CT data of these patients were imported into Mimics 21.0 software (Materialise, Leuven, Belgium), which is compatible with Digital Imaging and Communication in Medicine (DICOM) format, to generate three-dimensional images of the mandible. Subsequently, the reconstructed mandibles were saved as STL files (3D Systems, USA) for rapid printing.

### Design and production of occlusal alignment tablets

This study utilized an occlusal alignment piece designed by the researchers. It comprised three components: an electromagnetic sensor base, a connecting piece, and an occlusal splint. The electromagnetic sensor base connected the sensor, while the connecting piece featured a 2 mm diameter steel ball to aid in positioning. The occlusal splint was customizable to fit the mandibular dentition (refer to **Supplementary Figure 1**). It was essential for the occlusal splint to be easily removable or worn without abnormal wobbling, ensuring it maintained a consistent relative position to the mandible even under high-stress surgical conditions. This feature was crucial for fulfilling the requirements of clinical noninvasive alignment applications.

### Preoperative design

**Figure 3:**
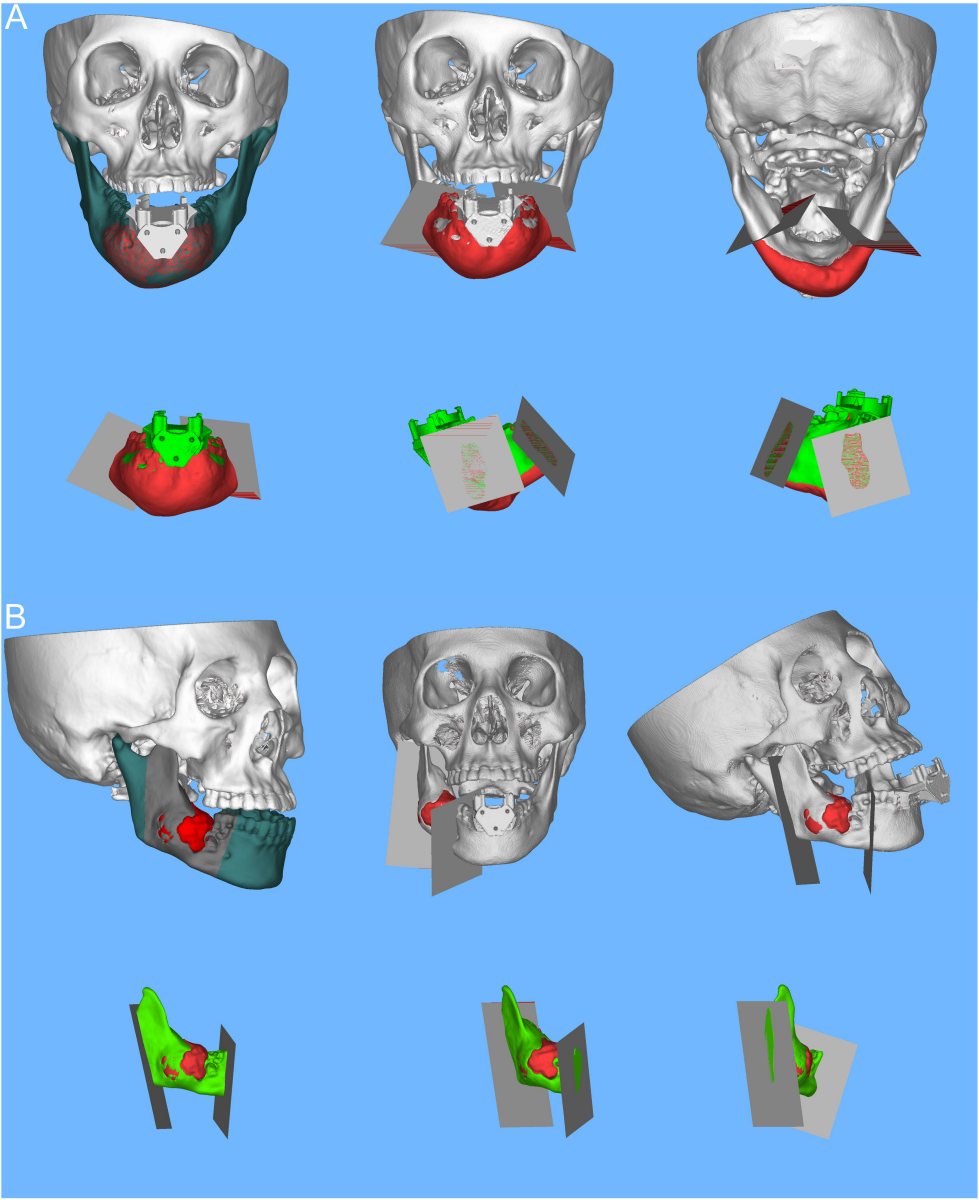
Preoperative mandibular tumor visualization in Mimics software (chin, ascending mandible). Design of mandibular tumor osteotomy planes. **(A)** Digital 3D reconstruction illustrating the extent of a chin tumor in a patient with a mandibular tumor; the osteotomy planes are tailored according to the extent of tumor invasion in the mandible, with a schematic representation of the osteotomized portion of the mandibular chin tumor in 3D. **(B)** Digital 3D reconstruction depicting the extent of a tumor in the ascending mandibular branch; based on the extent of tumor invasion in the mandible, the osteotomy plane is designed, accompanied by a schematic of the osteotomized portion of the tumor in the ascending mandible in 3D.

The customized alignment piece was affixed to the corresponding mandibular tumor model, and 3D-CT images were obtained to document the relative position information of the mandible to the alignment piece, which was then stored in DICOM format. Subsequently, the DICOM data were imported into Mimics software for 3D reconstruction. Utilizing the imaging data from patients with mandibular tumors, digital 3D reconstructions were performed to delineate the extent of the tumor. The preoperative design of the osteotomy plane for mandibular tumor patients was a collaborative effort between an experienced oral oncologist and an imaging surgeon. This design was based on the extent of tumor invasion in the mandible and was saved in STL format (see **Figures 3A, B**).

### Magnetic navigation robot registration and surgical path generation

**Figure 4:**
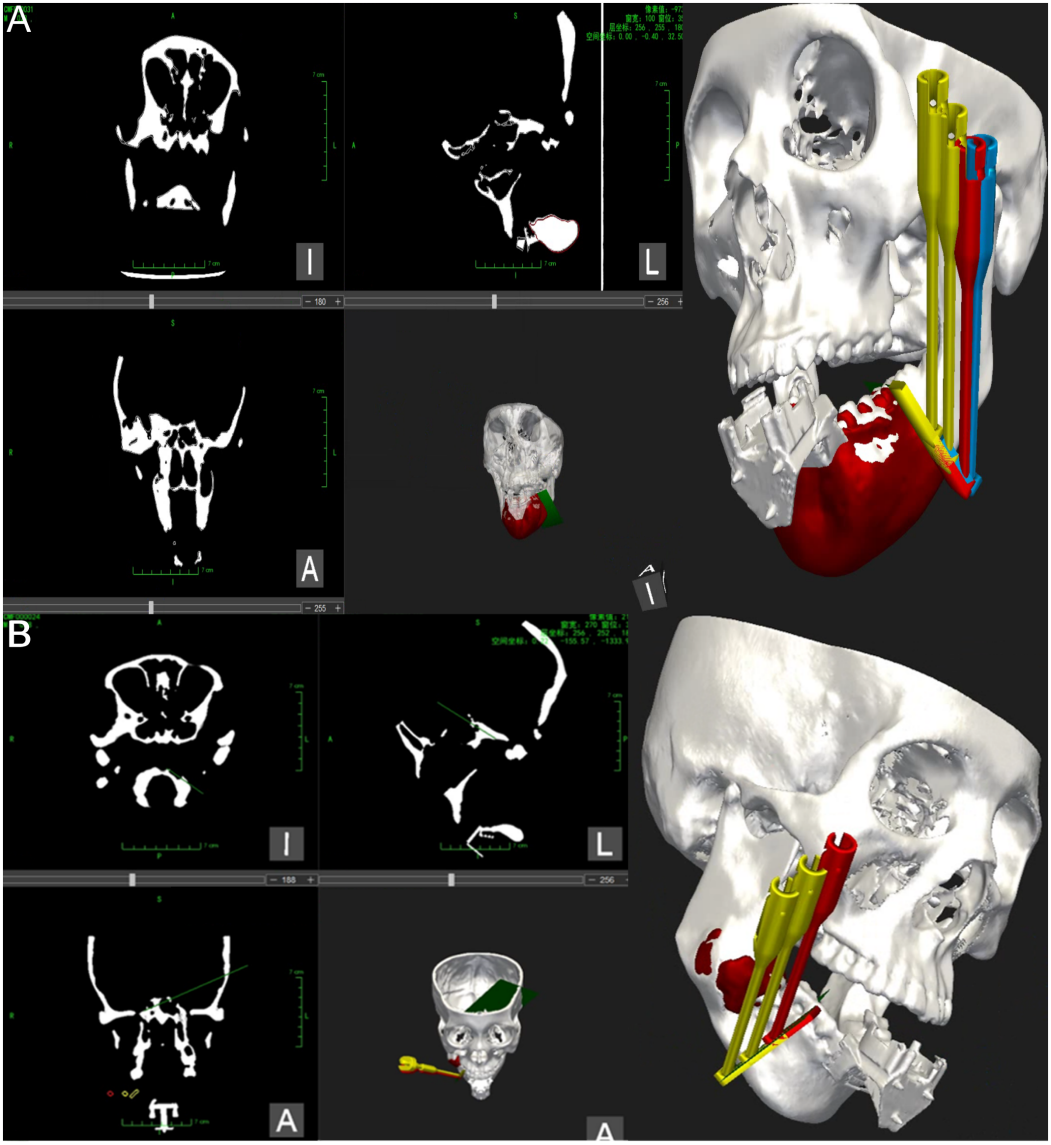
Magnetic navigation surgical operation system. **(A)** The operation interface of the mandibular tumor navigation and localization system software, illustrating the registration of the mandibular chin tumor model and relevant information regarding machine localization marker position. Additionally, the design of the robot end guide position and planning of the chin osteotomy path are depicted. **(B)** The operation interface of the mandibular tumor navigation and localization system software for registering the mandibular ascending tumor model and relevant information regarding machine localization marker position. Furthermore, the design of the robot end guide position and planning of the ascending mandibular osteotomy path are presented.

To ensure the stability of the mandibular tumor model during the procedure, a pneumatic arm was employed to secure the mandible onto the experimental table, maintaining it in a fixed position relative to the robot. Registration pieces were then appropriately affixed to the model, while any metal objects near the magnetic navigation electromagnetic sensors were removed to minimize electromagnetic interference. Subsequently, the preoperative design data was integrated into the craniomaxillofacial navigation and localization system. Alignment of the image with the model was achieved by identifying a steel ball with magnetic properties on the alignment piece. The robot was then aligned with the model by capturing the position information of the robot through the electromagnetic sensor within the same field. Following alignment, the system automatically generated a surgical path indicating the position of the robot’s end-motion target in the visualization interface of the software (see **Figures 4A, B**).

### Intraoral osteotomy of the mandibular tumor with robot-assisted navigation

During the surgical procedure, a headgear is positioned over the anatomical markers of the mandible and covered with plastic wrap to mimic the intraoperative view of the intraoral incision. Using a pull hook, the surgical assistant retracts the headgear to simulate the intraoral incision of the mandibular tumor, allowing the surgeon to perform mandibular debridement with a stripper. Once adequate exposure is ensured, an execution command is initiated, and the robot automatically moves the template to the planned osteotomy line. As the mandible is immobilized, the alignment piece can be removed before osteotomy, facilitating maneuverability in confined spaces. The template serves to guide the saw blade, with the operator holding the saw and executing the osteotomy on the lateral aspect of the mandible from posterior to anterior, following the angle of inclination of the template.

**Figure 5:**
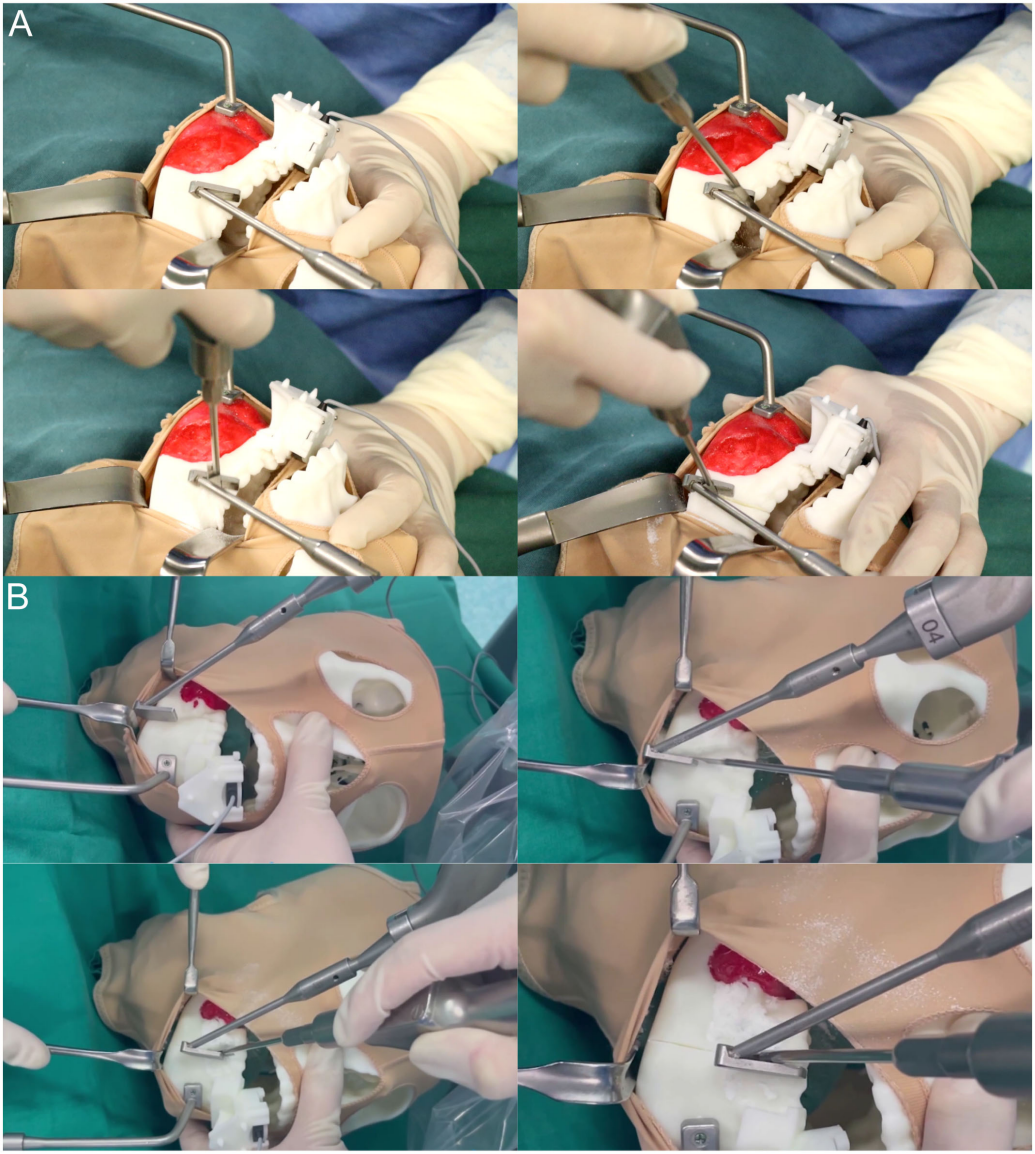
Magnetic navigation robot-assisted positioning osteotomy. **(A)** Following the preoperative setup of the magnetic navigation robot and the mandibular chin tumor model, osteotomy of the mandibular chin tumor model was performed under the positioning guidance of the magnetic navigation robot. At the same time, the control group underwent osteotomy without magnetic navigation guidance. **(B)** Following the preoperative setup of the magnetic navigation robot and the mandibular ascending tumor model, osteotomy of the mandibular ascending tumor model was performed under the positioning guidance of the magnetic navigation robot. At the same time, the control group underwent osteotomy without magnetic navigation guidance.

Throughout the osteotomy, the robot’s end-effector moves smoothly and uniformly along the mandibular surface along the planned path, providing real-time tracking and navigation (refer to **Figures 5A, B**).

### Postoperative osteotomy plane error analysis

**Figure 6:**
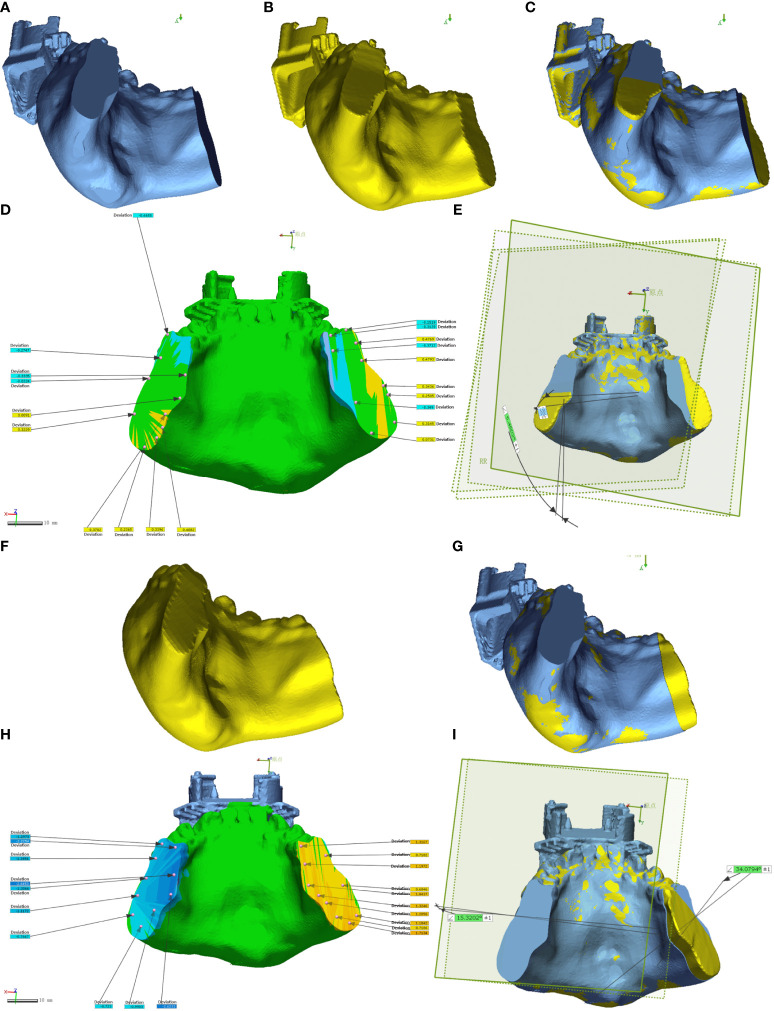
Geomagic Control software assesses errors in the osteotomy plane for mandibular chin tumors. **(A-E)** depict osteotomies guided by magnetic navigation robots, while **(F-I)** show unguided osteotomies.

**Figure 7:**
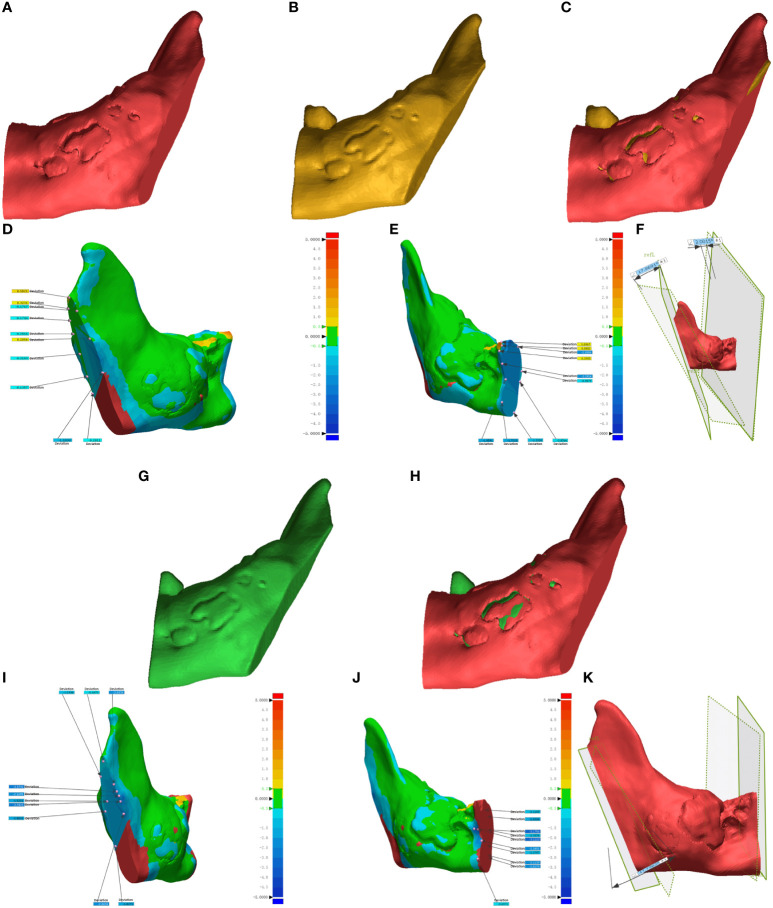
Geomagic Control software evaluates errors in the osteotomy plane for mandibular ascending branch tumors. **(A-F)** illustrate osteotomies guided by magnetic navigation robots, whereas **(G–K)** display unguided osteotomies.

The DICOM data from preoperative planning and the dissected mandible were processed using Mimics 21.0 for 3D reconstruction and saved in STL format. This data was then imported into Geomagic Control 2015 (3D Systems, Inc., USA) for reverse engineering. The pre-surgical design served as a reference (**Figures 6A**, **7A**), while post-surgical data served as test data (**Figures 6B**: EG, **F**: CG; **Figures 7B**: EG, **G**: CG). Optimal alignment was conducted to generate 3D color maps (**Figures 6C**: EG, **G**: CG; **Figures 7C**: EG, **H**: CG), showing the deviation of postoperative results from the preoperative design. In the fitted mandibular model, 10 points were randomly selected. Error-values between the preoperative design and actual osteotomy plane of the mandibular tumor were measured, with the average values recorded as locus errors for auto-aligned points (**Figures 6D** (EG), **Figures 7D, E** (EG); **Figure 6H**, (CG), **Figures 7I, J** (CG)). The accuracy of robot-assisted mandibular osteotomy was assessed by analyzing distance and angle error values between the preoperative design of the mandibular osteotomy plane and the actual osteotomy [**Figures 6E** (EG), **7F** (EG); **Figures 6I**, (CG), **7K** (CG)].

### Statistical analysis

The distribution of errors at various sites was assessed following automatic alignment using Geomagic Control software. To ensure the alignment accuracy met the evaluation criteria, a test value of “1” was established based on the inherent bias of the CT data, corresponding to a layer thickness of 1 mm, and a single sampling was conducted. A normality test was conducted on the measured data to determine the appropriate statistical test for positional and angular errors. A nonparametric test was employed if the data did not exhibit a normal distribution; otherwise, a t-test was utilized. Additionally, correlations between variables were analyzed using Pearson correlation analysis. A p-value of less than 0.05 was considered statistically significant. All statistical analyses were performed using R software (version 4.3.1).

## Results

### Error analysis of osteotomy planes

**Figure 8:**
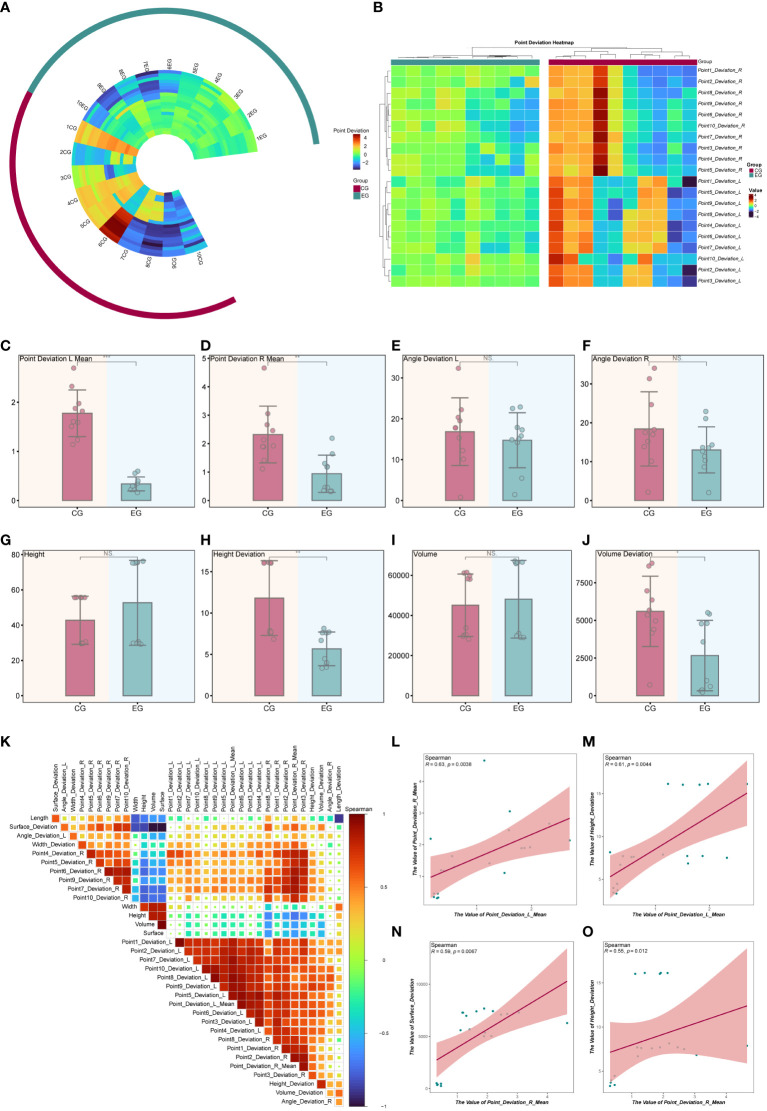
Statistical Analysis of Model Experimental Results. **(A)** Circular heatmap illustrating the baseline characteristics of the osteotomy plane point error. **(B)** The clustering heatmap provides further analysis of osteotomy plane point deviation, revealing differences in clustering between EG and CG groups. **(C-J)** The bar chart presents the statistical test of metrics between the EG and CG groups. **(K)** Heatmap depicting correlation analysis between the metrics in all the models. **(L-O)** Correlation scatter plot. *P < 0.05, **P < 0.01, ***P < 0.001.

Data from ten pairs of mandibular tumor models (5 pairs of mandibular chin and five pairs of mandibular ascending tumors) were analyzed. Preoperative design and automatic fitting of postoperative CT data were performed using Geomagic Control. Subsequently, 3D color deviation maps were generated to illustrate the disparities between the preoperative design and the dissected mandible. The baseline characteristics of point errors for the ten pairs of mandibular osteotomy planes were depicted using ring heatmaps (refer to **Figure 8A**), along with clustered heatmaps indicating significant differences in clustering between the point errors of the EG and CG groups (refer to **Figure 8B**). Baseline characterization results revealed no statistically significant differences in the length, width, height, volume, and surface area of the osteotomies between the EG and CG groups (see **Table 1**).

**Table 1:** Statistical analysis of baseline characteristics between EG and CG groups.

Variable	EG_mean	EG_sd	CG_mean	CG_sd	Method	p
Length (mm)	62.76	7.02	59.37	11.27	wilcox.test	0.449
Width (mm)	72.94	5.66	73.49	6.39	wilcox.test	0.739
Height (mm)	52.68	24.11	42.76	13.63	wilcox.test	0.307
Volume (mm3)	48093.78	19361.43	45095.00	15555.09	wilcox.test	0.481
Surface (mm2)	11261.89	4610.39	8880.02	1955.67	wilcox.test	0.481

In the EG group, the mean distance errors for the 10 randomly selected points on the left and right sides were 0.338 mm and 0.941 mm, respectively. In the EG group, the mean angular errors were 14.741 degrees for the left side section and 13.021 degrees for the right side. Conversely, in the CG, the mean distance errors were 1.776 mm and 2.320 mm for each of the 10 randomly selected points on the left and right sides, respectively. The mean angular errors were 16.841 degrees for the left side section and 18.416 degrees for the right side in the CG group. **Tables 2**,**3** present the statistical analysis of deviations in variables between the EG and CG groups.

**Table 2:** Statistical analysis (t.test) of deviations of variables between EG and CG groups.

Variable	EG: mean	EG: sd	CG: mean	CG: sd	Method	p
Point_Deviation_R_Mean (mm)	0.941	0.656	2.320	0.999	t.test	0.002
Angle_Deviation_L (°)	14.741	6.730	16.841	8.282	t.test	0.542
Angle_Deviation_R (°)	13.021	5.923	18.416	9.555	t.test	0.150
Width_Deviation (mm)	1.796	1.271	3.463	2.104	t.test	0.049

**Table 3:** Statistical analysis (wilcox.test) of deviations of variables between EG and CG groups.

Variable	EG: Median (Interquartile range)	CG: Median (Interquartile range)	Method	p
Point_Deviation_L_Mean (mm)	0.296 (0.238-0.387)	1.7111 (1.529-1.949)	wilcox.test	0.000
Length_Deviation (mm)	5.570 (4.180-6.818)	9.735 (4.700-13.133)	wilcox.test	0.151
Height_Deviation (mm)	5.580 (3.778-7.613)	11.955 (7.710-16.098)	wilcox.test	0.004
Volume_Deviation (mm3)	2287.980 (427.222-4810.673)	5519.685 (4554.100-6823.630)	wilcox.test	0.015
Surface_Deviation (mm2)	3746.200 (407.415-7279.635)	5970.845(5416.525-7136.983)	wilcox.test	0.481

In the EG group, the mean values of left and right-side point deviations were significantly lower than those in the CG group (refer to **Figures 8C, D**). In contrast, angular deviations did not show statistically significant differences between the two groups (refer to **Figures 8E, F**). Additionally, there were no statistically significant differences in height and volume between the EG and CG groups (refer to **Figures 8G, I**). However, compared to the CG group, the EG group exhibited significantly fewer height deviations (refer to **Figure 8H**) and smaller volume deviations (refer to **Figure 8J**). In summary, the osteotomy of mandibular tumors using the magnetic navigation robot demonstrated superiority over the control group in terms of both random point error and height and volume deviations. Furthermore, we analyzed two of the aforementioned metrics using spearman correlation (refer to **Figure 8K**). The results indicated that the mean values of left-side and right-side point deviation were positively correlated and statistically significant (refer to **Figure 8L**). In contrast, the mean values of left-side point deviation were positively correlated and statistically significant with height deviation (refer to **Figure 8M**). Moreover, the mean value of right-side point deviation was positively correlated and statistically significant with surface area deviation and height deviation (refer to **Figures 8N, O**). Additionally, we demonstrated the baseline characteristics of the modeled point deviations using other types of clustered heatmaps (refer to **Supplementary Figure 2A**). To better illustrate the differences in metrics between the EG and CG groups, we combined box-and-line and violin plots to depict the distributions of point deviation, angular deviation, height, height deviation, volume, and volume deviation across samples (refer to **Supplementary Figures 2B–I**).

To delve deeper into the correlation differences between the metrics within the EG and CG groups, correlation heatmaps and scatter plots were utilized for visualization. The findings indicated that the left-side point deviation exhibited a positive correlation with the right-side point deviation in the EG group, although it was not statistically significant. Similarly, the left-side point deviation positively correlated with height deviation, lacking statistical significance. However, the right-side point deviation demonstrated a positive correlation with both surface area deviation and height deviation, and these correlations were statistically significant (refer to **Supplementary Figure 3A**). In contrast, within the CG group, the right-side point deviation displayed a negative correlation with height deviation, which is not statistically significant (refer to **Supplementary Figure 3B**).

## Discussion

Mandibular tumors encompass a variety of benign and malignant tumors that develop in the craniomaxillofacial region (13). These tumors are categorized as odontogenic or non-odontogenic based on their tissue origin (14). Treatment principles for mandibular tumors parallel those for bone tumors in other locations, involving surgical resection (15, 16), as well as combination therapies such as chemotherapy and radiotherapy (17, 18). Tumor growth and invasion in this region can result in cosmetic deformities and impaired mastication, significantly impacting patients’ prognosis and quality of life. However, osteotomy of mandibular tumors via intraoral incision can notably minimize surgical trauma to soft tissues in the maxillofacial region, thereby reducing postoperative cosmetic defects. Achieving precise osteotomy during intraoral mandibular tumor procedures is essential, requiring accurate alignment with the tumor border. Nonetheless, conventional osteotomy methods often struggle to precisely control the intended osteotomy position and plane, resulting in excessive resection of normal bone tissue or residual tumor, adversely affecting postoperative quality of life and clinical outcomes. Consequently, achieving accurate positioning of the osteotomy line to match the preoperative design has remained a significant surgical challenge for many years.

Computer-aided design, virtual surgical planning, surgical modeling, rapid prototyping, intraoperative navigation, and other techniques have gained widespread usage (19–21). Surgical assistive guides have been employed to enhance surgical precision, reduce operative time, and lower complication rates (22, 23). However, challenges may arise during actual intraoperative scenarios with the use of surgical guides. Soft tissues such as ligaments, muscles, or mucous membranes may become trapped between the template and bone surface if the mandible is not adequately cleared. Also, the template might move horizontally or perpendicularly to the sagittal plane of the mandible because of tissue extrusion, or it might be hard to remove because of bleeding and instrument pressure, which could cause changes to the plan made before the surgery. The rapid advancement of digital technology has propelled the rapid growth and widespread adoption of robot-assisted techniques in various fields, owing to their precision, stability, and resistance to fatigue. Robot-assisted maxillofacial surgery has been shown to be safe, effective, and accurate in the past. However, because it relies on optical navigation systems, the surgery can only be done in well-lit areas with a wider field of view, which may not be ideal for mandibular tumor cases requiring intraoral incision surgery. The precise alignment and dynamic tracking of the robot’s coordinates with the target position during surgery pose significant challenges in the clinical application of robots. The electromagnetic navigation system represents a promising solution characterized by its compact size, independence from lighting conditions, and real-time tracking and localization capabilities. Sun et al. (7) initially demonstrated the feasibility of combining electromagnetic navigation technology with robot-assisted surgery in a surgical setting. However, to date, no experiments have been combining electromagnetic navigation with robot-assisted surgery for mandibular tumor procedures.

Using a robot-assisted surgical navigation system to get accurate localization during intraoperative osteotomy is what this study suggests should be done as a model surgical experiment for osteotomy for mandibular chin and ascending branch tumors. We employed an in-house robot with an electromagnetic navigation and localization system that amalgamates various technologies, including computer-aided design, three-dimensional image processing, and intraoperative electromagnetic navigation. This system made it easier to carry out motion commands inside a deep intraoral incision, which allowed for accurate localization during surgery. Previous studies have explored surgical navigation and robot-assisted techniques for mandibular angle osteotomy through model-based and animal experiments. At the same time, clinical trials have assessed the capabilities of augmented reality technology.

In this study, we effectively implemented an electromagnetically guided robotic surgical system in 10 sets of tumor osteotomies with intraoral incisions for mandibular tumors. This demonstrates the practicality and precision of system-based surgical assistance in localizing mandibular tumors with intraoral incisions. Our findings revealed that the accuracy of osteotomy in the experimental group surpassed that of the control group, where the surgeon determined the position based on preoperative planning and clinical expertise. The osteotomy site for intraoral incisions in mandibular tumors is often deep and obscured by soft tissues, prompting us to assess the utility of the robotic system in this context to ascertain its definitive role in intraoral surgery. To mimic the surgical scenario more accurately, we employed a mask with a brown elastic headgear to obscure the mandibular model, replicating the limited field of view encountered in actual surgical settings. The experimental results demonstrated significantly lower point error in the robotic group’s osteotomy plane position than the control group, indicating superior accuracy when utilizing the robotic system. The robotic system employed in this study utilizes electromagnetic navigation technology, offering the advantage of being noninvasive and convenient, as the use of an occlusal splint enables the alignment of the surgical plan with the natural environment. Automated positioning using small steel balls on positioning tablets simplifies the navigation process, reduces surgical exposure, and eliminates the inaccuracies associated with manually selecting positioning points.

Therefore, the outcomes of the model pilot study on mandibular tumors indicated that this protocol is straightforward and viable. However, no statistically significant distinction was observed in the osteotomy angle deviation between the experimental and control groups in this investigation, possibly due to the interference of metal or electrical equipment in the operating room with the magnetic navigation system. The experimental findings regarding mandibular tumors thus suggest that the robotic group still encounters a certain level of technical inaccuracies, which could stem from various sources: discrepancies between the model’s actual data and the results from CT scans; instability and uneven distribution of the magnetic field during magnetic navigation (e.g., interference from other metal objects in the magnetic field); registration inaccuracies; and/or minor shifts or deformations of the model during the experiment. To mitigate interference errors during magnetic navigation, some researchers have utilized simultaneous localization and mapping algorithms and preoperative calibration to reduce tracking errors in metallic environments effectively.

## Conclusions

Despite the commendable outcomes of our current study, our modeling experiments did not consider the influence of actual soft tissue factors, even when we used a brown elastic headgear to conceal the anatomical markings of the mandible and closely simulate the surgical environment. Also, getting around in the small space of an accurate intraoral incision is still hard because you have to avoid obstacles while positioning the robot, keep its end attached to nearby tissues while it’s moving, and look out for things that could get in the way of the operator’s view or the device’s closeness. Additionally, electrical devices in the operating room environment can interfere with the magnetic field of the robotic navigation system. Therefore, we need to conduct further research to create surgical instruments and beds made of materials that do not interfere with magnetic fields. It is imperative to continuously improve the system’s hardware and software in subsequent trials, such as by optimizing workflow to facilitate technology adoption and consolidating hardware facilities to reduce space requirements. Even though there are still problems and more applied research needs to be done, our results show that magnetic navigation techniques could be useful in model surgery for mandibular tumors because they are accurate, easy to use, and don’t require much damage.

## Data availability statement

The original contributions presented in the study are included in the article/**Supplementary material**, further inquiries can be directed to the corresponding author/s.

## Ethics statement

All procedures involved in the fabrication of the experimental model were conducted in compliance with ethical standards and guidelines. Written informed consent was obtained from the individual(s) for the publication of any identifiable images or data included in this article. Approval for the experimental protocols was obtainedfrom our independent ethical committee (Approval No. SH9H-2020-T267-3), and all methods were performed under relevant regulations and guidelines.

## Author contributions

ZZ: Writing – original draft. YZ: Writing – original draft. LL: Conceptualization, Writing – review & editing. WH: Conceptualization, Writing – original draft. CX: Conceptualization, Writing – review & editing. JL: Writing – review & editing. GC: Writing – review & editing.

## Supplementary material

Supplementary Figure 1 Self-aligning plastic on dental molds with occlusal registers, comprising sensor base, connecting part, occlusal splint, and steel positioning bead. **(A)** Front view; **(B)** Back view.

Supplementary Figure 2 Statistical Analysis of Model Experiment Results. **(A)** Clustered heatmap demonstrating the baseline characteristics of osteotomy plane point deviation. (b - I) Violin plot illustrating the statistical test of metrics between EG and CG groups, along with a box plot.

Supplementary Figure 3 Correlation Analysis of Model Experiment Results. **(A)** Heatmap and scatterplot of correlation analysis between metrics within the EG group. **(B)** Heatmap and scatterplot of correlation analysis between metrics within the CG group.
