# Supplementary figures and images for: Intelligent electromagnetic navigation system for robot-assisted intraoral osteotomy in mandibular tumor resection: a model experiment
Source: Front Immunol. 2024 Jul 25;15:1436276. doi: 10.3389/fimmu.2024.1436276 (PMC11306084; doi:10.3389/fimmu.2024.1436276)

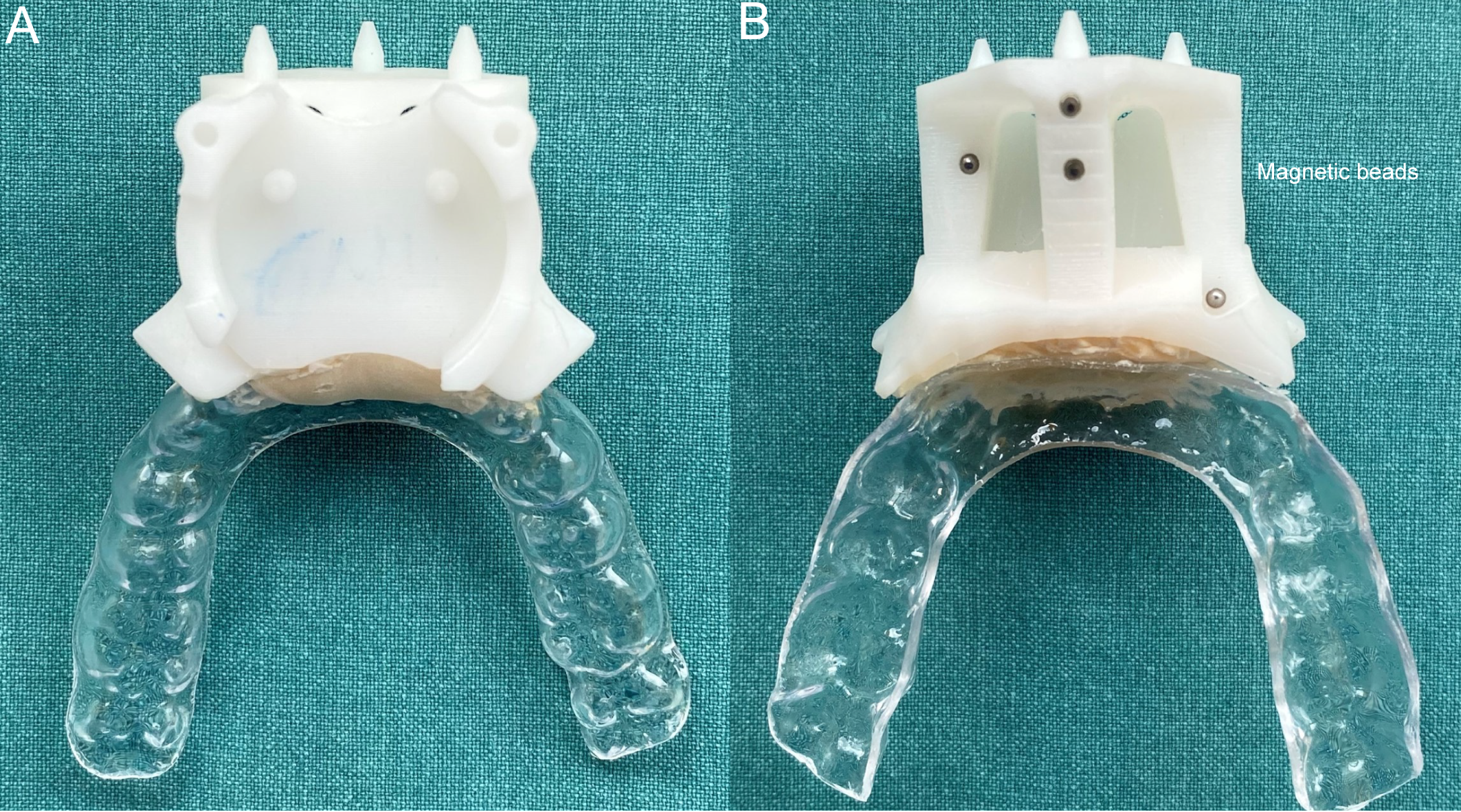

Supplement: Supplementary Figure 1 — Self-aligning plastic on dental molds with occlusal registers, comprising sensor base, connecting part, occlusal splint, and steel positioning bead. (A) Front view; (B) Back view. [file Image_1.tif]

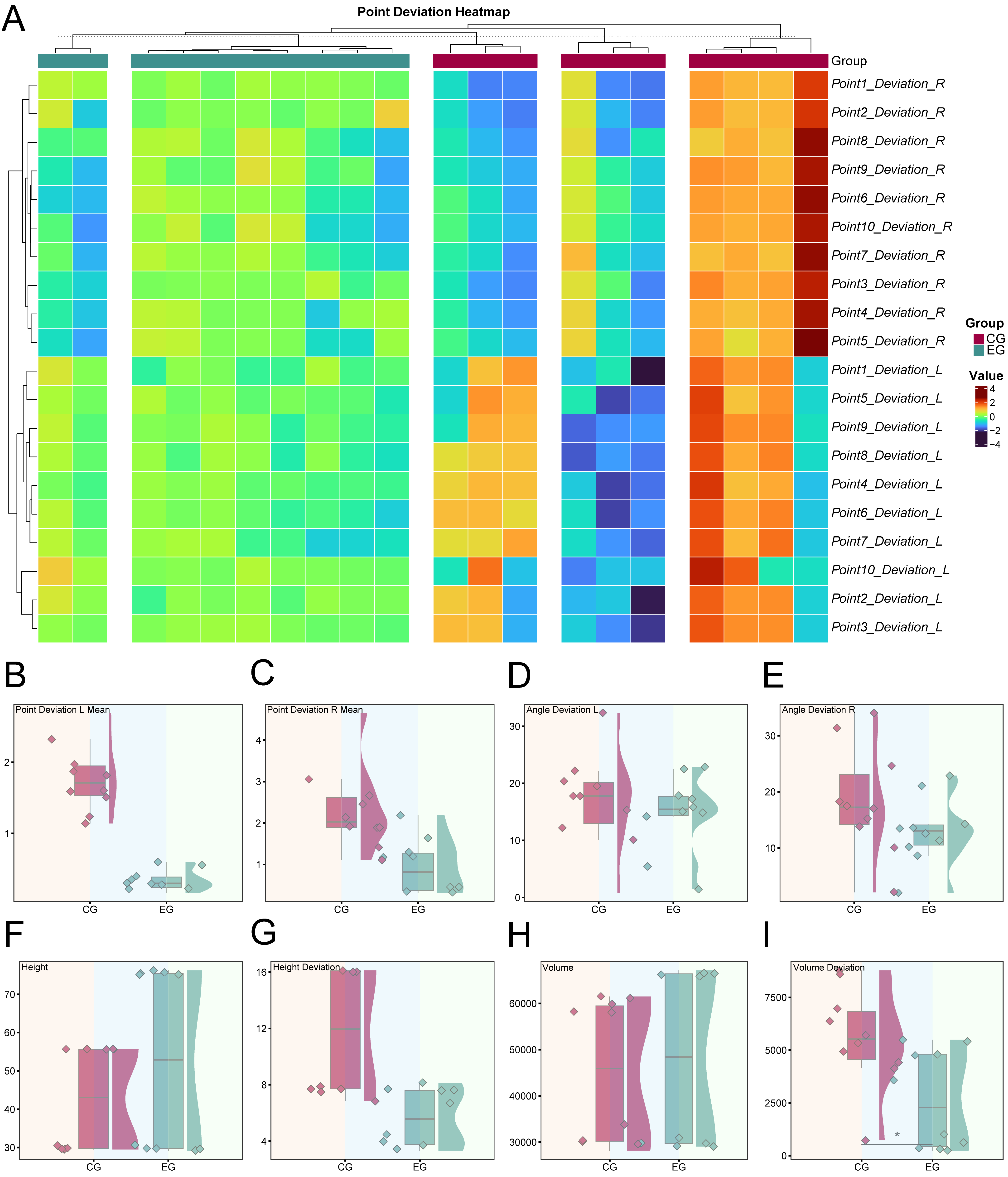

Supplement: Supplementary Figure 2 — Statistical Analysis of Model Experiment Results. (A) Clustered heatmap demonstrating the baseline characteristics of osteotomy plane point deviation. (b - I) Violin plot illustrating the statistical test of metrics between EG and CG groups, along with a box plot. [file Image_2.tif]

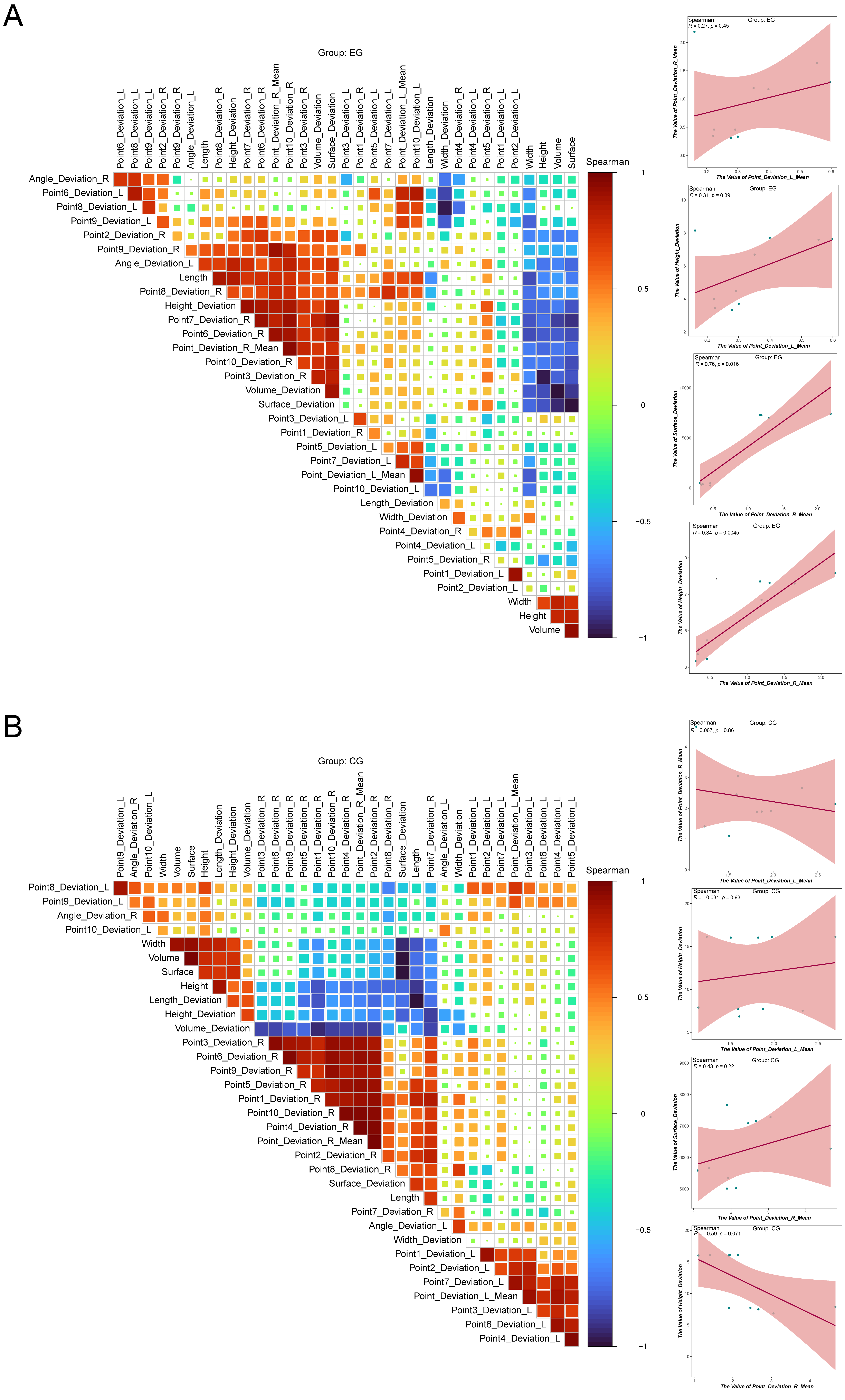

Supplement: Supplementary Figure 3 — Correlation Analysis of Model Experiment Results. (A) Heatmap and scatterplot of correlation analysis between metrics within the EG group. (B) Heatmap and scatterplot of correlation analysis between metrics within the CG group. [file Image_3.tif]
